# Relationship Between Critically Longer Working Hours, Depressive Symptoms, and Suicidal Ideation Among Obstetricians and Gynecologists in Japan

**DOI:** 10.3390/healthcare12232364

**Published:** 2024-11-26

**Authors:** Masatoshi Ishikawa, Ryoma Seto, Michiko Oguro, Yoshino Sato

**Affiliations:** 1 Research Institute, Tokyo Healthcare University, Higashi Gotanda, Shinagawa City, Tokyo 141-0022, Japan; r-seto@thcu.ac.jp (R.S.); m-oguro@thcu.ac.jp (M.O.); 2 Okoge Co., Ltd., Higashiikebukuro, Tokyo 170-0013, Japan; 10yoshinosato05@gmail.com

**Keywords:** *Karōshi*, working hour, depressive symptoms, suicide ideation, Japan

## Abstract

**Background/Objectives:** The Ministry of Health, Labor and Welfare in Japan has been promoting physicians’ working style reforms since 2019. This study aimed to update the relationship between working hours, depressive symptoms, and suicidal ideation among obstetricians and gynecologists, based on the physicians’ working style reforms. **Methods:** A questionnaire-based survey was conducted among obstetricians and gynecologists, and valid responses were obtained from 1164 physicians. Multivariable logistic regression analysis was performed to identify significant associations. **Results:** Of the respondents, 49.8% were female, and most physicians were aged 30–39 (32.1%). Precisely, 57.4% worked in public hospitals, and 47.9% worked in urban areas. Physicians working 40 ≤ x < 60 h per week accounted for the largest proportion of physicians. Depressive symptoms and suicidal ideation accounted for 16.4% and 3.6% of participants, respectively. The following factors were significantly associated with depressive symptoms as a dependent variable: other occupation, having two or three children, working 60–80 h or >100 h per week, and working in rural areas. None of these variables was significantly associated with suicidal ideation. **Conclusions:** The physicians’ working style reforms have reduced the number of working hours for obstetricians and gynecologists. However, rates of depressive symptoms and suicidal ideation have not improved.

## 1. Introduction

The number of working hours among laborers in Japan is relatively high compared to international levels [1]. A comparison between occupations shows that physicians have particularly long working hours [2]. Among physicians, those working in obstetrics and gynecology hospitals tend to work longer hours [3].

In 2016, the Ministry of Health, Labor and Welfare established the Study Group on Physicians’ Working Style Reform, which investigated measures to promote physicians’ working style reforms and published a report in 2019 [4]. The report states that the maximum overtime hours for physicians will begin in April 2024, starting at 960 h per year for Level A and 1860 h per year for Level B (provisional special level for securing regional medical care). It outlines concrete measures to reduce working hours, including specific regulations on physicians’ overtime, proper management of working hours, utilization of existing industrial health systems, and promotion of task shifting.

There is a wide range of national and international evidence regarding the adverse health effects of long working hours. According to a systematic review by Bannai et al., long working hours are associated with depressive state, anxiety, sleep conditions, and coronary heart disease [5]. Suicide due to depression caused by excessive workload and death from ischemic heart disease and cerebrovascular disease are called “*Karōshi*” or “overwork death” and have become a public health issue unique to China and other East Asian countries [6].

Medicine is a stressful profession, and residents are especially prone to depression and burnout [7,8,9]. According to a review of factors associated with physician burnout, younger age, female sex, negative marital status, long working hours, and low reported job satisfaction are predictors of burnout [10]. Long working hours negatively affect residents’ health and medical safety [11,12].

In 2019, we conducted a study that, for the first time, revealed the extent of overwork among obstetricians and gynecologists in Japan and demonstrated that long working hours among physicians may lead to depressive symptoms and suicidal ideation [13].

Following the implementation of the physicians’ working style reforms in 2019, it was hoped that working hours would be reduced, leading to a decrease in the proportion of doctors suffering from depression or suicidal thoughts. However, no studies have examined the improvements in depressive symptoms and suicidal ideation after the reform.

This study aimed to update the understanding of the relationship between working hours, depressive symptoms, and suicidal ideation among obstetricians and gynecologists in light of the physicians’ working style reforms implemented in 2019.

## 2. Materials and Methods

### 2.1. Participants

We selected 1170 hospitals across Japan with obstetrics and gynecology departments whose hospital names were disclosed through the hospital bed function reporting system [14]. In November 2023, we sent survey request forms to managers and physicians of the obstetrics and gynecology departments. An online questionnaire survey was conducted, taking approximately 10 min to complete, and responses were collected via Google Forms. No special follow-up was conducted, and respondents who did not complete the survey were excluded. In addition, no incentives or compensation were offered to participants.

### 2.2. Measurements

#### 2.2.1. Depressive Symptoms

To assess the respondents’ depressive state, we used the Japanese version (QIDS-SR-J) [15] of the 16-Item Quick Inventory of Depressive Symptomatology Self-Report Version (QIDS-SR) [16], used worldwide to assess the severity of depression and defined depression, as moderate or high on the scale. The QIDS-SR is a 16-item self-administered rating scale that evaluates depression severity and aligns with the diagnostic criteria for major depressive disorder in the DSM-IV of the American Psychiatric Association [16]. The QIDS-SR-J is the validated Japanese version of this tool.

We also determined whether those who were depressed also had suicidal ideation, which tended to be higher among physicians in previous studies [17]. As in previous studies, we considered as having suicidal ideation those who responded as follows: “I think about suicide or death for several minutes several times a week” and “I have thought about suicide or death in great detail several times a day or made specific suicide plans or actually attempted to die”.

#### 2.2.2. Working Hours and Risk Factors

We first described the physician attributes (sex, age, occupation, weekly working hours, foundational entity of the workplace, total number of hospital beds, and region) for all the respondents and for those with and without depressive symptoms, with the cutoff set at moderate or higher on the QIDS-SR-J (Table 1). Age was classified into six groups: <30 years, 30s, 40s, 50s, 60s, and ≥70 years. The foundational entities of the workplace were classified into four groups: public, national universities, private universities, and private (excluding private universities). Occupation was classified into four groups: department manager, staff, specialized physician, and others. The weekly working hours were classified into five groups: x < 40 h, 40 ≤ x < 60 h, 60 ≤ x < 80, 80 ≤ x < 100 h, and x ≥ 100 h. The total number of hospital beds was classified into five groups: x < 200 beds, 200 ≤ x < 400 beds, 400 ≤ x < 600 beds, 600 ≤ x < 800 beds, and x ≥ 800 beds. In Japan, 344 secondary medical areas were classified into three categories based on the combination of population size and population density in 2023: the first group (urban), the second group (intermediate), and the third group (rural) [18].

**Figure 1:**
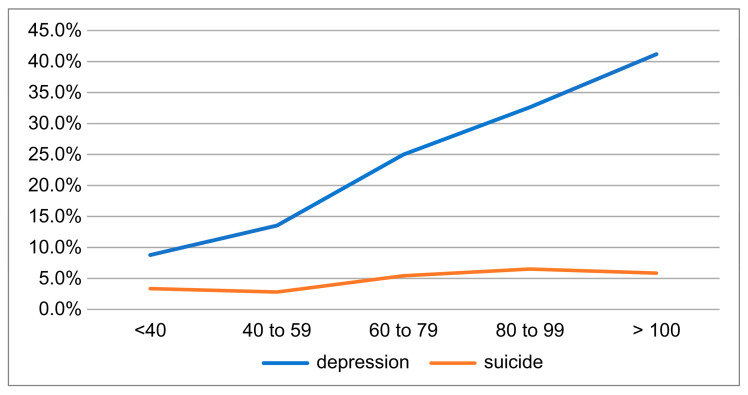
Relationship between working hours and depression symptoms/suicide ideation.

Figure 1 shows the percentage of depressive symptoms and suicidal ideation for each category of working hours per week.

### 2.3. Statistical Analysis

To examine the relationship between depressive symptoms, working hours, and other risk factors among obstetricians and gynecologists, we performed a multivariable logistic regression analysis, with the cutoff set at moderate or higher on the QIDS-SR-J (Table 2). Depressive symptoms were set as the dependent variable, and physician attributes (sex, age, occupation, weekly working hours, whether to perform delivery, number of children, foundational entity of the workplace, total number of beds, and region) were set as independent variables. We also conducted a multivariate logistic regression analysis with suicidal ideation as the dependent variable and the other variables as independent variables (Table 2). The QIDS-SR16 has proven to be a popular choice given that it was developed to align with the Diagnostic and Statistical Manual of Mental Disorders-IV criteria and is quick and simple to administer [19]. The required sample size for logistic regression analysis is typically 10 times the number of explanatory variables (9), thus requiring a minimum of 90 samples; our sample size of 1164 is therefore sufficient [20].

Statistical analyses were considered significant at *p*-values < 0.05. STATA 17.0 was used for all statistical analyses.

### 2.4. Ethical Considerations

This study was approved by the Human Research Ethics Committee of the Tokyo Healthcare University (approval number: Education 023-03B). The study objective and measures to ensure secure data management were stated on the first page of the questionnaire. We also explained to the potential participants that their involvement in the study was voluntary. Informed consent was obtained from all participants. The results were analyzed separately from personal information to ensure anonymity and confidentiality of personal information.

## 3. Results

Questionnaires were sent to 1170 hospitals across Japan, and valid responses were received from 423 hospitals (valid response rate: 36.2%) and 1164 physicians. The 1164 OBGYNs who responded accounted for 16.3% of the 7127 hospital-based obstetricians and gynecologists in the 2020 Statistics on Physicians, Dentists, and Pharmacists conducted by the Ministry of Health, Labor and Welfare [21].

As shown in Table 1, 49.8% of the respondents were female, and most physicians were aged 30–39, accounting for 32.1% of the population. A total of 57.4% of the physicians worked in public hospitals, and 47.9% worked in urban areas.

Physicians working 40 ≤ x < 60 h per week accounted for the largest proportion of physicians (61.6%), followed by 20.6% in the 60–79 h per week group, 12.7% in the <40 h per week group, 4.0% in the 80 ≤ x < 100 h per week group and 1.5% in the x ≥ 100 h per week group. In our study in 2019, physicians working 60–80 h per week accounted for the largest proportion of physicians (37.5%), followed by 27.1% in the x ≥ 100 h per week group and 19.5% in the 80–100 h/week group.

Precisely 16.4% of respondents were rated as having moderate-to-severe depression on the QIDS-SR-J. As shown in Figure 1, the proportion of patients with depressive symptoms was higher in the group that worked longer hours. It was 25.0% in the 60 ≤ x < 80 h per week group and 32.6% in the 80 ≤ x < 100 h per week group. Suicidal ideation was present in 3.6% of respondents and was higher in the group with 80–100 h per week (6.5%).

Table 2 shows the results of the multivariate logistic regression analysis. With depressive symptoms as the dependent variable, the following showed significant associations: other occupation (control: department manager); having two children (control: no children); having three children (control: no children); 60–80 h per week (control: <40 h per week); 80–100 h per week (control: <40 h per week); >100 h per week (control: <40 h per week); and rural areas (control: urban).

In the analysis with suicidal ideation as the dependent variable, no significant association was found for any of the variables.

## 4. Discussion

In this study, physicians working > 60 h per week accounted for 26.0% of the respondents, revealing the excessive workload of obstetricians and gynecologists working in Japanese hospitals. Weekly working hours of 60 h correspond to 4 h of overtime work per day compared to the legal working hours of 8 h per day and 40 h per week, as stipulated by the Labor Standards Act, and the number of overtime hours exceeds approximately 80 h per month. Such long working hours are referred to as “death-from-overwork level” since they are strongly associated with the development of mental disorders and cardiovascular diseases due to psychological stress, and they meet the certification criteria for occupational hazards [22,23]. Since 49.1% of the respondents seem to have working conditions that exceed “*Karōshi*’s criteria”, there is an urgent need to improve the working environment for obstetricians and gynecologists in the development of a sustainable obstetric and gynecological medical system.

In contrast, 84.1% of physicians in the 2019 survey worked over 60 h per week; thus, working hours were found to have decreased considerably in the current survey [13]. This may be due to progress in physicians’ working style reforms, such as task shifting. However, the Ministry of Health, Labor and Welfare has given guidance that hours for “physician’s study” are not to be added to working hours, which was unclear in 2019. Additionally, the hours that were previously considered working hours have been made clear to employees as a result of the acquisition of “the permission of night and day shifts”, and much of the time during night and day shifts is now considered to be break time. These points may have impacted the above comparison [24,25].

There are a few points to note regarding whether obstetricians and gynecologists really work fewer hours. First, the definition of a physician’s study time has been defined by the Ministry of Health, Labor, and Welfare; however, in some cases, the distinction between labor and self-study is not always straightforward [26]. Additionally, the following can be said regarding “the permission of night and day shifts” and whether the workload during the on-call day shift is greater or less than that during the night and day shift. In a study of Japanese obstetricians and gynecologists, 17% answered that their workload was very heavy, and 31% answered that their workload was somewhat heavy; thus, it should be noted that the criteria for night and day shift permits may be loose [27].

Precisely 16.4% of respondents were rated as having moderate-to-severe depression on the QIDS-SR-J, and suicidal ideation was present in 3.6% of respondents, which was approximately the same as in the 2019 survey (16.4% for depressive symptoms and 3.3% for suicidal ideation). In other words, there was no improvement in the rates of depressive symptoms and suicidal ideation despite the reduction in working hours compared with 2019. As pointed out in the previous paragraph, this may be related to the fact that the definition of working hours has changed so that the actual time spent in the hospital has not changed or to factors other than working hours, such as people relationships [28].

Compared to previous studies, a study of employed Japanese physicians found the prevalence of depressive symptoms to be 6.5% and suicidal ideation to 3.6%, both lower than the results of this study [17]. One possible reason for this is that the working hours of obstetricians and gynecologists are longer than those of other medical departments [3]. There are no other studies on obstetricians and gynecologists worldwide, with the exception of ours; therefore, it is difficult to make comparisons.

In multiple logistic regression analysis with depressive symptoms as the dependent variable, depressive symptoms were significantly lower for those with two or three children (control: no children) and significantly higher for those who worked 60–80 h/week, 80–100 h/week, more than 100 h/week (control: under 40 h/week), and rural areas (control: urban areas). The odds ratio for an 80–100 h per h/week was the highest among the independent variables. In the analysis with suicidal ideation as the dependent variable, no significant association was found for any of the variables.

According to previous studies, younger physician age, female sex, negative marital status, and long working hours have been said to be predictors of burnout [10]. A previous study conducted in 2019 showed a significant positive association between depressive symptoms and negative marital status, long working hours, being male, and aged 30–49 years. In the present study, long working hours were significantly associated with depressive symptoms; however, the other factors were not consistent with those in previous studies. Having children may reduce the likelihood of depressive symptoms, possibly because of reduced working hours resulting from time spent on childcare and education.

Regarding the relationship between depressive symptoms and factors such as the characteristics of the hospital where the physicians work and the region in which the hospital is located, only rural areas were significantly associated with the independent variables in this study. Physician shortages are a problem in underpopulated areas, leading to longer working hours, which can lead to depression [18].

Among obstetricians and gynecologists, physicians who perform deliveries have longer working hours and lower career satisfaction [29]. A more detailed survey of physicians’ practice would enable a more elaborate discussion.

### Study Limitations

This study had some limitations. First, only 423 of the 1170 hospitals (36.2%) and 1164 physicians participated in the study, which is a relatively small number because participation was voluntary. It is possible that physicians who responded to our questionnaire were interested in issues such as working conditions, mental health, or depressive symptoms. Additionally, severely depressed physicians may not have responded to the survey.

Second, we used a self-administered questionnaire and defined residents who had a QIDS-SR-J score of ≥16 as screening-positive for depressive symptoms. In addition, the QIDS-SR is based on DSM-IV criteria rather than DSM-5, so this algorithm may not fully align with current diagnostic standards or detect all cases of depressive symptoms. For assessing suicidal ideation, quantitative measures such as the Suicide Intent Scale were not used, which may limit the assessment’s thoroughness.

Third, the relationship between working hours, depressive symptoms, and suicidal ideation was statistical rather than causal. This may be related to factors that were not measured, such as job satisfaction, support systems, underlying diseases, alcoholism, economic background, and sleep status [28].

Fourth, in defining suicidal ideation, this study did not examine details such as the full spectrum of ideation severity, frequency, and intent—as were performed in previous studies—which remains an area for future research [30].

Fifth, with regard to working hours, responses were provided in 20 h increments to increase the response rate. Using narrower categories or continuous analysis of hours could have yielded more precise insights.

## 5. Conclusions

Following a study conducted in 2019, this study sheds light on the relationship between overwork and mental health among obstetricians and gynecologists in Japan. Although working hours decreased, the rates of depressive symptoms and suicidal ideation have remained unchanged, suggesting that factors other than working hours, as well as changes in the definition of working hours, may contribute to depressive symptoms and suicidal ideation. Physician working-style reforms need to be vigorously promoted to improve the mental health of obstetricians and gynecologists.

## Figures and Tables

**Table 1:** Characteristics of participants.

	Total		Without Depressive Symptoms		Depressive Symptoms		Suicide Ideation	
Total number of participants, n	1164		973		191		42	
% of all participants	100.0%		83.6%		16.4%		3.6%	
Sex, n, %								
Female	580	49.8%	475	48.8%	105	55.0%	25	59.5%
Male	584	50.2%	498	51.2%	86	45.0%	17	40.5%
Age, n, %								
<30	130	11.2%	104	10.7%	26	13.6%	7	16.7%
30–39	374	32.1%	301	30.9%	73	38.2%	18	42.9%
40–49	318	27.3%	263	27.0%	55	28.8%	9	21.4%
50–59	187	16.1%	162	16.6%	25	13.1%	5	11.9%
60–69	141	12.1%	130	13.4%	11	5.8%	2	4.8%
≧70	14	1.2%	13	1.3%	1	0.5%	1	2.4%
Occupation								
Department manager	325	27.9%	291	29.9%	34	17.8%	9	21.4%
Staff	582	50.0%	477	49.0%	105	55.0%	20	47.6%
Specialized physician	223	19.2%	179	18.4%	44	23.0%	12	28.6%
Other	34	2.9%	26	2.7%	8	4.2%	1	2.4%
Delivery-related work								
Yes	1060	91.1%	880	90.4%	180	94.2%	37	88.1%
No	104	8.9%	93	9.6%	11	5.8%	5	11.9%
Number of children								
None	409	35.1%	319	32.8%	90	47.1%	20	47.6%
1	189	16.2%	152	15.6%	37	19.4%	6	14.3%
2	363	31.2%	320	32.9%	43	22.5%	10	23.8%
3	168	14.4%	150	15.4%	18	9.4%	4	9.5%
≥4	35	3.0%	32	3.3%	3	1.6%	2	4.8%
Working hours per week, n, %								
<40	148	12.7%	135	13.9%	13	6.8%	5	11.9%
40–59	710	61.0%	614	63.1%	96	50.3%	20	47.6%
60–79	240	20.6%	180	18.5%	60	31.4%	13	31.0%
80–99	46	4.0%	31	3.2%	15	7.9%	3	7.1%
≧100	17	1.5%	10	1.0%	7	3.7%	1	2.4%
Entity of employer								
Public	668	57.4%	565	58.1%	103	53.9%	21	50.0%
National University	121	10.4%	91	9.4%	30	15.7%	3	7.1%
Private University	69	5.9%	55	5.7%	14	7.3%	5	11.9%
Private	306	26.3%	262	26.9%	44	23.0%	13	31.0%
Employer’s total no. of beds								
<200 beds	90	7.7%	80	8.2%	10	5.2%	4	9.5%
≥200–<400 beds	320	27.5%	271	27.9%	49	25.7%	9	21.4%
≥400–<600 beds	399	34.3%	340	34.9%	59	30.9%	14	33.3%
≥600–<800 beds	203	17.4%	163	16.8%	40	20.9%	6	14.3%
≥800 beds	152	13.1%	119	12.2%	33	17.3%	9	21.4%
Area, n, %								
Urban	558	47.9%	472	48.5%	86	45.0%	21	50.0%
Intermediate	489	42.0%	412	42.3%	77	40.3%	18	42.9%
Rural		10.1%	89	9.1%	28	14.7%	3	7.1%

**Table 2:** Logistic regression analysis of factors associated with depressive symptoms and suicidal ideation.

Depressive Symptoms	OR	95% CI	*p*-Value	Suicidal Ideation	OR	95% CI	*p*-Value
Sex				Sex			
Female	Reference			Female	Reference		
Male	1.05	0.73–1.51	0.80	Male	0.72	0.3–1.48	0.37
Age				Age			
<30 years	Reference			<30 years	Reference		
30 s	1.13	0.58–2.19	0.72	30 s	0.97	0.28–3.37	0.96
40 s	1.35	0.64–2.88	0.43	40 s	0.49	0.11–2.20	0.36
50 s	1.06	0.44–2.56	0.89	50 s	0.29	0.47–1.79	0.18
60 s	0.68	0.24–1.92	0.46	60 s	0.21	0.02–1.80	0.16
≥70 years	0.82	0.09–7.86	0.87	≥70 years	1.62	0.11–24.48	0.73
Occupation				Occupation			
Department manager	Reference			Department manager	Reference		
Staff	1.34	0.78–2.31	0.29	Staff	0.73	0.24–2.23	0.58
Specialized physician	1.13	0.54–2.37	0.74	Specialized physician	0.61	0.14–2.74	0.52
Other	3.06	1.08–8.62	0.04	Other	0.63	0.06–6.34	0.69
Delivery-related work				Delivery-related work			
No	Reference			No	Reference		
Yes	1.39	0.69–2.78	0.36	Yes	0.69	0.22–2.16	0.53
Number of children				Number of children			
None	Reference			None	Reference		
1	0.75	0.46–1.22	0.26	1	1.00	0.38–2.67	0.98
2	0.47	0.29–0.75	0.00	2	1.90	0.33–2.17	0.74
3	0.44	0.24–0.83	0.01	3	2.37	0.25–3.05	0.84
≥4	0.39	0.11–1.38	0.14	≥4	2.14	0.52–14.67	0.24
Working Hours per Week				Working Hours per Week			
<40 h per week	Reference			<40 h per week	Reference		
40~60 h per week	1.43	0.76–2.70	0.27	40~60 h per week	1.00	0.34–2.91	1.00
60~80 h per week	2.51	1.26–5.00	0.01	60~80 h per week	1.90	0.60–6.06	0.28
80~100 h per week	6.88	2.88–16.45	0.00	80~100 h per week	2.37	0.48–1168	0.29
≥100 h per week	5.93	1.80–19.57	0.00	≥100 h per week	2.15	0.21–21.92	0.52
Foundational Entity of Employer				Foundational Entity of Employer			
Public	Reference			Public	Reference		
National University	1.43	0.77–2.64	0.25	National University	0.58	0.15–2.29	0.44
Private University	0.87	0.36–2.06	0.74	Private University	0.60	0.13–2.72	0.50
Private	1.08	0.69–1.70	0.74	Private	1.29	0.57–2.92	0.54
Employer’s Total No. of Beds				Employer’s Total No. of Beds			
<200 beds	Reference			<200 beds	Reference		
≥200–<400 beds	0.92	0.41–2.03	0.83	≥200–<400 beds	0.54	0.14–2.01	0.36
≥400–<600 beds	1.03	0.46–2.31	0.94	≥400–<600 beds	0.84	0.23–3.06	0.80
≥600–<800 beds	1.20	0.50–2.92	0.68	≥600–<800 beds	0.63	0.13–3.00	0.56
≥800 beds	1.62	0.62–4.20	0.32	≥800 beds	2.03	0.44–9.38	0.37
Workplace				Workplace			
Urban	Reference			Urban	Reference		
Intermediate	1.16	0.81–1.67	0.41	Intermediate	0.93	0.47–1.85	0.84
Rural	1.97	1.11–3.49	0.02	Rural	1.06	0.33–3.43	0.93

*p* < 0.05. OR, odds ratio; CI, confidence interval.

## Data Availability

Some data are unavailable due to ethical restrictions.
